# Association of serum 25-hydroxyvitamin D with cardiovascular and all-cause mortality in patients with chronic kidney disease: NHANES 2007‒2018 results

**DOI:** 10.1016/j.clinsp.2024.100437

**Published:** 2024-07-11

**Authors:** Luohua Li, Jinhan Zhao

**Affiliations:** a Department of Nephrology, Jiujiang City Key Laboratory of Cell Therapy, Jiujiang No. 1 People’s Hospital, Jiujiang, China; b The Third Unit, The Department of Hepatology. Beijing Youan Hospital, Capital Medical University, Beijing, China; c Beijing Institute of Hepatology, Beijing Youan Hospital, Capital Medical University, Beijing, China

**Keywords:** 25(OH)D, Cardiovascular Diseases, All-Cause Mortality, NHANES, CKD

## Abstract

**Background:**

Vitamin D insufficiency is a prevalent issue in patients suffering from CKD. The purpose of this study was to determine whether serum 25(OH)D levels are associated with all-cause and cardiovascular mortality in patients with CKD.

**Methods:**

To examine the associations between 25(OH)D levels and cardiovascular mortality, this retrospective cohort study used the National Health and Nutrition Examination Survey (NHANES) and the National Death Index (NDI) 2007‒2018 database. A total of 2,668 eligible subjects were included in this study, with follow-up conducted until December 31, 2019. The associations were assessed using Cox proportional hazards regression, restricted cubic splines, Kaplan-Meier survival curves, and competing risks survival analysis. Furthermore, subgroup and sensitivity analyses were performed.

**Results:**

During a median follow-up of 72 months in a weighted population of 11,715,452 eligible participants, there were 665 deaths from any cause, including 196 cardiovascular-related deaths. After adjusting for covariates, lower levels of 25(OH)D were significantly associated with increased risks for both all-cause mortality (HR= 0.85, 95 % CI 0.77∼0.94) and cardiovascular mortality (SHR= 0.80, 95 % CI 0.67∼0.94). Consistent results were also observed when analyzing 25(OH)D as a categorical variable (quartile). Compared to group Q1, both group Q3 (HR = 0.71, 95 % CI 0.54‒0.93) and group Q4 (HR = 0.72, 95 % CI 0.55‒0.94) exhibited a significantly reduced mortality risk. Weighted restricted cubic splines revealed an inverse J-shaped linear association between levels of 25(OH) D and all-cause mortality ((PNonliner > 0.05). Subgroup analysis and sensitivity analysis yielded similar findings.

**Conclusions:**

All-cause mortality and cardiovascular disease-related mortality were significantly increased by lower 25(OH)D levels, both as continuous and categorical variables. 25(OH)D has an inverse J-shaped linear association with all-cause and cardiovascular mortality.

## Highlights

• The NHANES data was utilized in this study for conducting a comprehensive long-term follow-up analysis.
• Deficiency of 25(OH)D was associated with an increased risk of all-cause mortality among patients with CKD.
• Deficiency of 25(OH)D is associated with an increased risk of cardiovascular mortality in patients with CKD.

## Introduction

Chronic Kidney Disease (CKD) is an important global public health problem. The incidence of CKD has increased significantly due to factors such as hypertension, metabolic diseases, and aging. It affects approximately 8 %‒16 % of the world's population and is the 16th leading cause of death.^1^ The metabolite 25-hydroxyvitamin D (25(OH)D) plays an important role in the human body. It is converted into its active form in the kidneys and helps maintain mineral metabolism balance and regulate the immune system. Disorders of vitamin D metabolism are common in patients with CKD and are routinely evaluated and treated in this population. However, some studies have shown that lower vitamin D concentrations are associated with an increased risk of mortality and cardiovascular complications in patients with CKD.^2^, ^3^, ^4^ In a recent cohort study, researchers found no significant association between initial 25(OH)D levels and CKD outcomes through multivariate analysis.^5^ The relationship between vitamin D deficiency and prognosis in patients with chronic kidney disease is controversial. Furthermore, there is no consensus on optimal serum levels of 25-hydroxyvitamin D in patients with chronic kidney disease. Given the growing evidence for the importance of 25(OH)D in the development of CKD, it is crucial to investigate whether baseline 25(OH)D levels can serve as a predictor of mortality in a large group of CKD patients. Such studies will provide valuable insights into the pathogenesis of CKD and provide additional evidence to support the development of preventive and therapeutic strategies. This study used data from the 2007‒2018 National Health and Nutrition Examination Survey (NHANES) to explore the association between all-cause and Cardiovascular Disease (CVD) mortality and 25(OH)D levels in adults with CKD in the United States.

## Subjects and methods

### Subjects

**Fig. 1:**
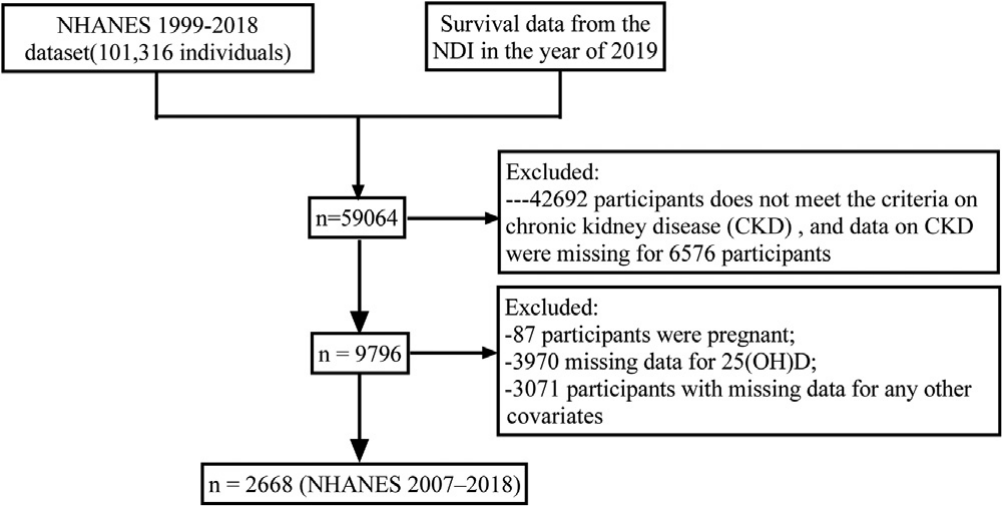
Flow chart of enrolled patients.

The NHANES is a biennial cross-sectional survey conducted by the National Center for Health Statistics (NCHS) in the United States. This ongoing survey aims to evaluate the health and nutritional status of both adults and children, providing a nationally representative sample of the civilian population. The assessment of mortality and its potential causes was conducted through the linkage of death records, spanning from the date of interview until the date of death or review, which concluded on December 31, 2019. This study encompassed all individuals aged 18 years or older, while excluding pregnant participants during examination and those with incomplete data. Furthermore, individuals with inadequate mortality follow-up data were also excluded. Ultimately, a total of 2668 eligible participants from the NHANES spanning from 2007 to 2018 were included in this study (Fig. 1). The data used in this study come from a de-identified public database (https://www.cdc.gov/nchs/nhanes/index.htm [accessed on August 11, 2023]).^6^

According to the KDIGO guidelines, the stages of CKD were determined based on estimated Glomerular Filtration Rate (eGFR) and/or evidence of renal damage. Stage 1 is defined as eGFR ≥ 90 mL/min/1.73 m^2^ and Urine Albumin-to-Creatinine Ratio (UACR) ≥ 30 mg/g. Stage 2 is characterized by an eGFR of 60‒89 mL/min/1.73 m^2^ and a UACR ≥30 mg/g. Stage 3 is indicated by an eGFR of 30‒59 mL/min/1.73 m^2^. Stage 4 is marked by an eGFR of 15‒29 mL/min/1.73 m^2^. Stage 5 is identified by an eGFR < 15 mL/min/1.73 m^2^.^7^ The NHANES study was conducted by the Centers for Disease Control and Prevention (CDC) and approved by the National Center for Health Statistics (NCHS) Ethical Review Board. All participants provided informed consent.^8^

### Study variables

The variables in the present study included sex (male and female), age, ethnicity (eth), Body Mass Index (BMI), medical history of Cardiovascular Disease (CVD), hypertension, diabetes, education level (below high school, high school graduate or equivalent, and above high school), BMI measurements, questionnaire responses regarding self-reported hypertension and diabetes status, as well as physician-diagnosed CVD. Laboratory data including Hemoglobin (Hb), Alanine Aminotransferase (ALT), Aspartate Aminotransferase (AST), Total bilirubin (Tb), Alkaline Phosphatase (ALP), albumin, Creatinine (Cr), Uric Acid (UA), Blood Urea Nitrogen (BUN), Phosphorus (P), Calcium (Ca.), High-Density Lipoprotein cholesterol (HDL-c), Low-Density Lipoprotein cholesterol (LDL-c), eGFR, UACR, dietary vitamin D intake, and C-Reactive Protein (CRP) were collected through standardized interviews. Physical examinations, laboratory tests, and questionnaires were administered by trained medical personnel. The concentration of serum 25(OH)D has been determined using standardized Liquid Chromatography-tandem Mass Spectrometry (LC–MS/MS) since the 2007–2008 cycle. Following CDC recommendations, the authors utilized LC–MS/MS equivalent data. The details of the conversion are specified elsewhere. From NHANES 2007–2010, the 2.5^th^–97.5^th^ percentile of 25-hydroxyvitamin D levels in the population over 1 year was 23.5–124 nmoL/L, the arithmetic mean was 68.0 nmoL/L.^9^ Several factors, including season, race (skin darkness), latitude, sun protection behaviors, diet, and supplement intake, have been identified as influencing the levels of 25(OH)D. The observed variation in 25(OH)D values due to seasonal changes in ultraviolet radiation underscores the significance of individual sunlight exposure.^10^^,^^11^ The highest levels of 25(OH)D are typically detected during the summer to fall months, while the lowest levels occur during late winter and early spring.

### Ascertainment of outcomes

All-cause mortality was defined as death resulting from any cause during follow-up based on the records of the National Death Index prior to December 31, 2019. CVD mortality was identified as per the International Classification of Diseases, Tenth Revision codes I00–I09, I11, I13, I20‒I51, or I60–I69.^12^^,^^13^

### Statistical analysis

To account for the sophisticated and complex probability sampling technique employed in the NHANES surveys, particular subgroups of individuals from the non-institutionalized civilian population in the United States were deliberately oversampled.^6^ To ensure the reliability of these results, the authors included the sample weights produced through NHANES analyses. Descriptive statistics were employed to examine the attributes and distribution of the population. Continuous variables were presented as mean ± standard error when normally distributed and as medians or interquartile ranges for skewed distributions. Categorical variables were displayed as frequencies and percentages. 25(OH)D was categorized according to quartiles, and p-values for trend were determined. Survival estimates and cumulative event rates were compared using both the Kaplan-Meier method and the competing risk model. Cox proportional hazard models estimated Hazard Ratios (HR) and 95 % Confidence Intervals (95 % CI) for investigating the relationship between 25(OH)D and all-cause, as well as CVD mortality. Sensitivity analyses were conducted, excluding participants who received dialysis within the last 12 months to diminish potential reverse-causation bias. Additionally, stratification was conducted according to age (< 60 years vs. ≥ 60 years), gender (male vs female), BMI (< 25, 25‒30, ≥ 30), race, hypertension history (yes or no), diabetes history (yes or no), and Cardiovascular disease history (CV) to investigate the correlation between 25(OH)D and all-cause mortality. Potential interactions between 25(OH)D and different stratification factors were investigated. Statistical analyses were performed using R software (version 4.3.1). Two-sided p-values < 0.05 were considered statistically significant.

## Results

### Population characteristics

A total of 2668 participants (NHANES 2007–2018) met the eligibility criteria, resulting in a weighted nationally representative total of 11,715,452 participants. A total of 665 deaths occurred during the 16,551 person-years of follow-up, including 196 from cardiovascular disease. Among these deaths, women accounted for 54.69 % and the average age was 55.24±0.46 years old. The median follow-up period was 6 years. The weighted mean ± standard error of 25(OH)D was 60.7± 0.8 nmoL/L. Baseline characteristics according to 25(OH)D quartiles are shown in Table 1. Participants with lower 25(OH)D levels were more likely to be female and non-Hispanic black. Moreover, they had a higher percentage of hypertension. In addition, they had higher BMI levels.

**Table 1:** Baseline characteristics of patients with CKD according to quartiles of 25(OH)D in NHANES 2007–2018.

25(OH)D (nmoL/L)	Total (unweighted n= 2668)	Q1 (≤ 45.90) (unweighted n= 670)	Q2 (45.90 to ≤ 58.50) (unweighted n= 667)	Q3 (58.50 to ≤ 78.60) (unweighted n= 670)	Q4 (> 78.60) (unweighted n= 661)	p-value
Weighted, n	11715452	2616264	2943814	2999357	3156017	
Age (years)	55.24± 0.46	54.93± 0.90	54.05±0.77	55.08± 0.91	56.76± 0.80	0.2
Sex (%)						<0.001
Female	1394 (54.69)	383 (59.97)	364(58.48)	352 (55.47)	294 (45.84)	
Male	1274 (45.31)	286 (40.03)	303 (41.52)	318 (44.53)	367 (54.16)	
Ethnicity (%)						<0.001
Mexican American	496 (13.17)	118 (13.86)	136 (14.27)	130 (13.58)	112 (11.27)	
Non-Hispanic Black	830 (20.61)	299 (35.29)	241 (23.95)	160 (14.63)	130 (11.18)	
Non-Hispanic White	823 (51.83)	137 (37.76)	173 (48.71)	226 (54.16)	286 (63.89)	
Other Hispanic	266 (6.57)	64 (6.22)	53 (5.08)	86 (8.94)	63 (6.01)	
Other Race	253 (7.82)	52 (6.87)	64 (7.99)	68 (8.69)	70 (7.64)	
Citizenship status (%)						0.22
Not a citizen of the US	377 (11.11)	74 (8.96)	103 (12.36)	104 (12.63)	96 (10.45)	
Citizen by birth or naturalization	2283 (88.64)	594 (91.04)	562(87.64)	562 (87.37)	564 (89.55)	
Marriage (%)						0.02
Divorced	340 (12.95)	87 (13.01)	85 (12.32)	81 (13.71)	87 (14.13)	
Living with partner	163 (6.80)	43 (6.20)	35 (6.98)	47 (9.04)	38 (5.70)	
Married	1185 (47.59)	251 (42.47)	303 (48.33)	298 (47.90)	332 (55.31)	
Never married	351 (13.83)	125 (19.35)	88 (14.73)	82 (12.70)	56 (10.94)	
Separated	120 (3.46)	42 (5.71)	29 (3.53)	27(3.17)	22 (2.17)	
Widowed	417 (12.77)	95 (13.27)	107 (14.11)	110 (13.49)	105 (11.75)	
Hypertension (%)						0.01
No	856 (36.35)	189 (29.55)	211 (34.83)	239 (40.25)	217 (39.85)	
Yes	1812 (63.65)	480 (70.45)	456 (65.17)	431 (59.75)	444 (60.15)	
Diabetes (%)						0.08
No	1482 (58.99)	351 (54.17)	367 (56.94)	378 (60.80)	385 (62.96)	
Yes	1186 (41.01)	318 (45.83)	300 (43.06)	292 (39.20)	276 (37.04)	
Cardiovascular history (%)						0.18
No	1925 (75.19)	467 (72.80)	492 (78.74)	491 (79.27)	475 (77.69)	
Yes	653 (22.25)	176 (27.20)	156 (21.26)	156 (20.73)	164 (22.31)	
Education level (%)						0.04
< High School	444 (11.02)	93 (8.98)	107 (10.81)	128 (12.21)	116(11.88)	
High school	512 (16.33)	155(21.21)	127 (15.96)	111 (15.41)	119 (13.70)	
➢	High School	1707 (72.51)	421 (69.81)	430 (73.23)	430 (72.38)	425 (74.42)
BMI (kg/m²)	31.55± 0.24	32.37± 0.40	31.92 ± 0.52	31.60 ± 0.54	30.50 ± 0.42	0.01
Follow-up time, months	72.75± 1.27	73.28± 2.40	72.50 ± 2.46	74.01± 1.81	71.70 ± 2.53	<0.001
WBC (10^9/L)	7.73± 0.10	7.53± 0.10	8.05 ± 0.32	7.62 ± 0.10	7.70 ± 0.11	<0.001
Hb (g/dL)	13.81± 0.05	13.42± 0.10	13.82 ± 0.09	13.82 ± 0.06	14.13 ± 0.10	<0.001
ALT (U/L)	25.66± 0.79	27.78± 3.12	26.50± 1.33	24.61 ± 0.81	24.17 ± 0.58	<0.001
AST (U/L)	26.70± 0.61	30.16± 2.41	27.33 ± 1.15	25.14 ± 0.53	24.78 ± 0.45	<0.001
Tb (umoL/L)	11.24± 0.13	11.52± 0.32	11.17± 0.27	10.77± 0.23	11.55± 0.27	<0.001
ALP (mmoL/L)	76.54± 0.76	79.21± 1.36	76.91± 1.44	78.48± 2.22	72.17± 1.06	<0.001
Albumin (g/L)	41.17± 0.10	40.12± 0.20	41.25 ± 0.20	41.37 ± 0.16	41.93 ± 0.17	<0.001
Creatinine (umoL/L)	95.55± 2.36	96.96± 3.20	96.83 ± 5.04	92.39 ± 2.83	95.90 ± 4.49	<0.001
UA (umoL/L)	350.54± 2.71	357.45± 4.42	347.67±6.10	346.19±4.74	351.86±5.16	<0.001
BUN (mmoL/L)	5.81± 0.08	5.77± 0.19	5.72± 0.17	5.68± 0.13	6.01± 0.16	<0.001
Phosphorus (mmoL/L)	1.21± 0.01	1.20± 0.01	1.22± 0.01	1.20± 0.01	1.19± 0.01	<0.001
Calcium (mmoL/L)	2.34± 0.00	2.32± 0.01	2.34± 0.01	2.34± 0.01	2.35± 0.00	<0.001
HDL-c (mmoL/L)	1.29± 0.01	1.36± 0.03	1.28± 0.02	1.27± 0.02	1.27± 0.02	<0.001
LDL-c (mmoL/L)	2.83± 0.03	2.87± 0.07	2.76± 0.07	2.92± 0.07	2.79± 0.06	0.28
eGFR (mL/min/1.73 m^2^)	82.98± 0.92	83.60± 1.73	85.00± 1.64	83.09± 1.72	80.75± 1.61	<0.001
UACR (mg/g)	268.95± 25.59	415.36± 78.13	294.51± 51.41	223.74± 32.35	171.50± 22.90	0.004
Dietary Vitamin D (mcg)	8.50± 0.28	6.43± 0.34	8.26±0.51	9.07± 0.57	9.67± 0.57	<0.001
CRP (mg/L)	0.28(0.12, 0.67)	0.37(0.13, 0.75)	0.29(0.14,0.66)	0.23(0.12, 0.57)	0.27(0.13, 0.64)	0.21

Values are weighted mean ± SE for continuous variables or weighted % for categorical variables. BMI, Body Mass Index; WBC, White Blood Cell; Hb, Hemoglobin; ALT, Alanine Aminotransferase; AST, Aspartate Aminotransferase; Tb, Total Bilirubin; ALP, Alkaline Phosphatase; UA, Uric Acid; BUN, Blood Urea Nitrogen; HDL-c, High-Density Lipoprotein cholesterol; LDL, Low-Density Lipoprotein; e-GFR, Estimated Glomerular Filtration Rate; UACR, Urinary Albumin-to-Creatinine Ratio; CRP, C-Reaction Protein.

### The relationship between 25(OH)D and the prognosis of CKD

**Fig. 2:**
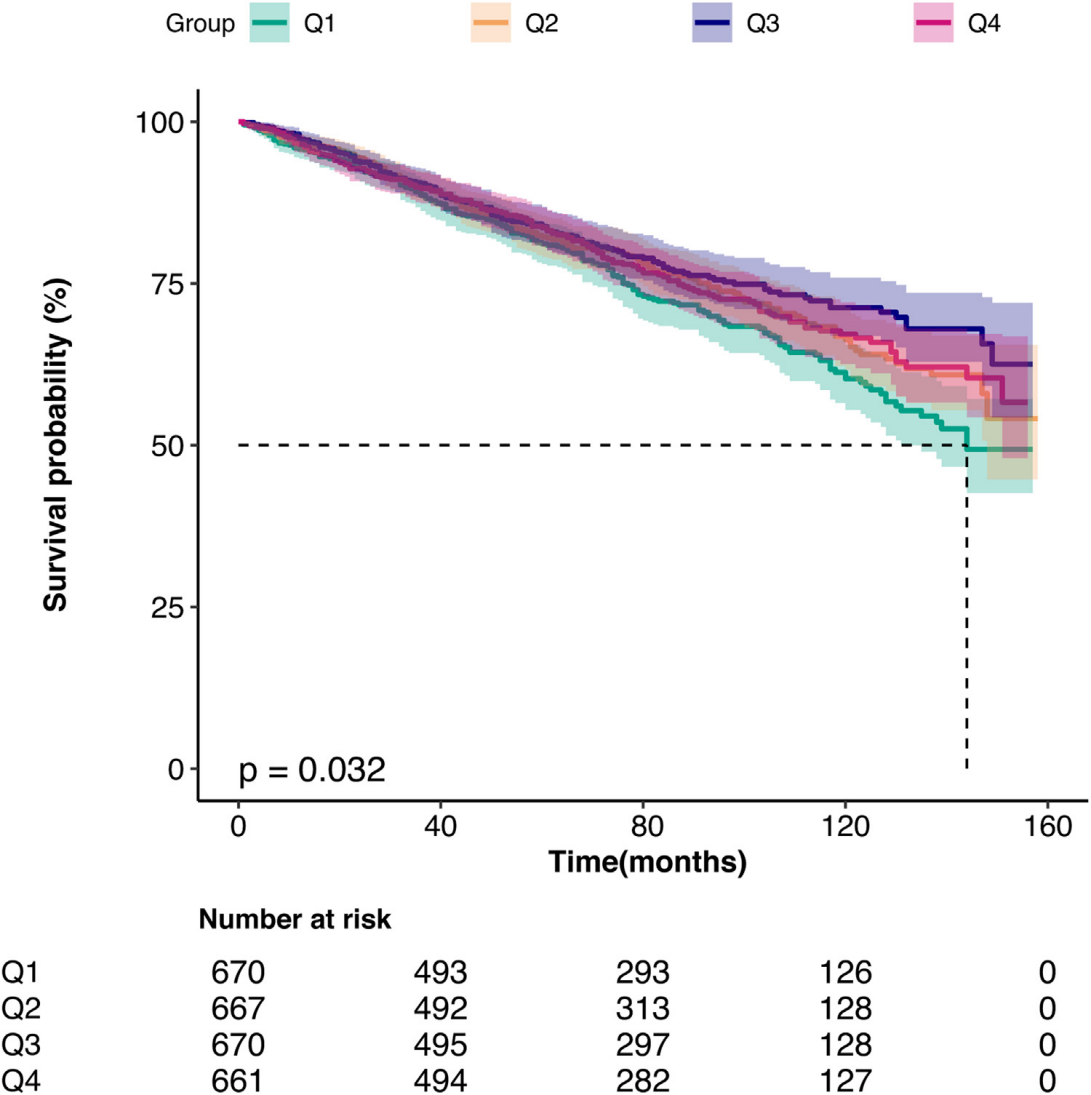
Kaplan-Meier analysis of all-cause death in 25(OH)D quartiles (log-rank, p=0.032)

**Fig. 3:**
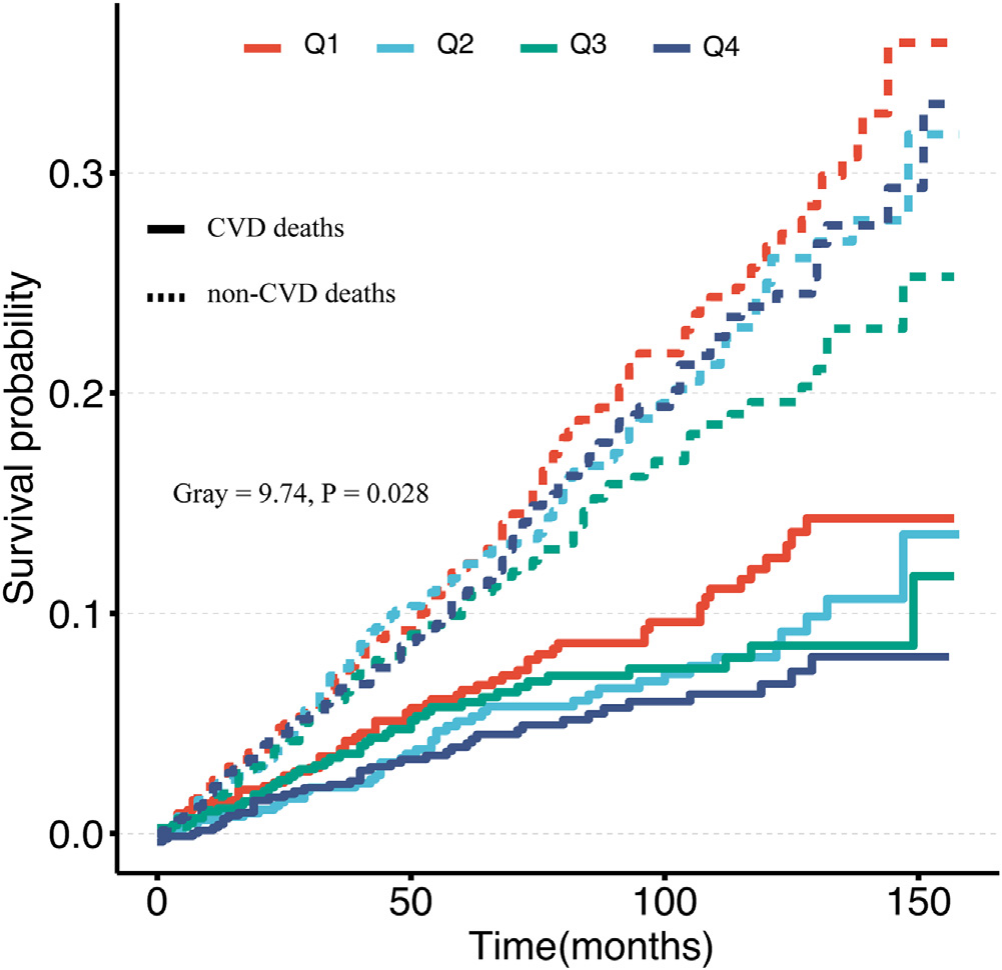
Cumulative incidence of CVD deaths for the competing risks analysis.

Kaplan-Meier survival analysis and competing risk model showed a significant increase in the risk of all-cause and cardiovascular death in individuals with lower 25(OH)D levels compared with those with higher levels (Fig. 2, Fig. 3). Weighted multivariable Cox regression analysis, after adjustment for age, sex, race, BMI, ethnicity, presence of chronic diseases, and laboratory parameters, showed a strong association between lower 25(OH)D levels and all-cause mortality. This association was also found to be significantly associated with increased cardiovascular mortality (Table 2). The multivariable-adjusted HR and 95 % CI for all-cause mortality decreased progressively from the lowest to the highest 25(OH)D categories (≤ 45.9, 45.9‒58.5, 58.5‒78.6, and > 78.6), with a trend p-value of < 0.05. Similarly, the corresponding SHR and 95 % CI for cardiovascular death also showed a decreasing trend across 25(OH)D categories, with a significant trend p-value of < 0.05 (Table 3). Furthermore, each standard deviation increase in 25(OH)D levels was associated with a 15 % lower risk of all-cause death and a 20 % lower risk of cardiovascular death.

**Table 2:** Association between 25(OH)D and all-cause mortality (Cox regression model) (n= 2668).

25(OH)D	Crude Model		Model 1		Model 2	
	HR (95% CI)	p-value	HR (95% CI)	p-value	HR (95% CI)	p-value
Per SD increment	0.92 (0.85, 0.99)	0.022	0.82 (0.76, 0.89)	<0.001	0.85 (0.77, 0.94)	0.001
Categories						
Q1	Reference	Reference	Reference			
Q2	0.83 (0.68, 1.02)	0.084	0.84 (0.68, 1.04)	0.116	0.9 (0.7, 1.16)	0.423
Q3	0.73 (0.59, 0.9)	0.004	0.63 (0.5, 0.79)	<0.001	0.71 (0.54, 0.93)	0.013
Q4	0.83 (0.67, 1.02)	0.08	0.64 (0.51, 0.79)	<0.001	0.72 (0.55, 0.94)	0.014
p for trend	0.036	<0.001	0.006			

Crude Model: Unadjusted.

Model 1: Adjust for sex, age, eth, BMI, history of cardiovascular disease, hypertension and diabetes.

Model 2: Adjust for sex, age, eth, BMI, smoke, history of cardiovascular disease, hypertension, diabetes, Hb, ALT, AST, Tb, ALP, Albumin, Creatinine, UA, BUN, Phosphorus, Calcium, HDL-c, LDL-c, eGFR, UACR, Dietary Vitamin D, CRP.

**Table 3:** Association between 25(OH)D and Cardiovascular Disease (CVD) mortality (Fine-Gray competing risk model).

25(OH)D	Crude Model		Model 1		Model 2	
	SHR (95% CI)	p-value	SHR (95% CI)	p-value	SHR (95% CI)	p-value
Per SD increment	0.79 (0.68∼0.92)	0.003	0.80 (0.69∼0.92)	0.002	0.80 (0.67∼0.94)	0.005
Categories						
Q1	Reference	Reference	Reference			
Q2	0.68 (0.45∼1.02)	0.06	0.69 (0.47∼1.01)	0.057	0.67 (0.33∼1.35)	0.26
Q3	0.66 (0.44∼1)	0.051	0.65 (0.44∼0.96)	0.029	0.52 (0.25∼1.07)	0.076
Q4	0.52 (0.34∼0.79)	0.002	0.55 (0.37∼0.82)	0.003	0.47 (0.22∼0.98)	0.044
p for trend	0.003	0.004	0.055			

Crude Model: Unadjusted.

Model 1: Adjust for sex, age, eth, BMI, history of cardiovascular disease, hypertension and diabetes.

Model 2: Adjust for sex, age, eth, BMI, smoke, history of cardiovascular disease, hypertension, diabetes, Hb, ALT, AST, Tb, ALP, Albumin, Creatinine, UA, BUN, Phosphorus, Calcium, HDL-c, LDL-c, eGFR, UACR, Dietary Vitamin D, CRP.

### Dose-response association between 25(OH)D and CKD mortality

**Fig. 4:**
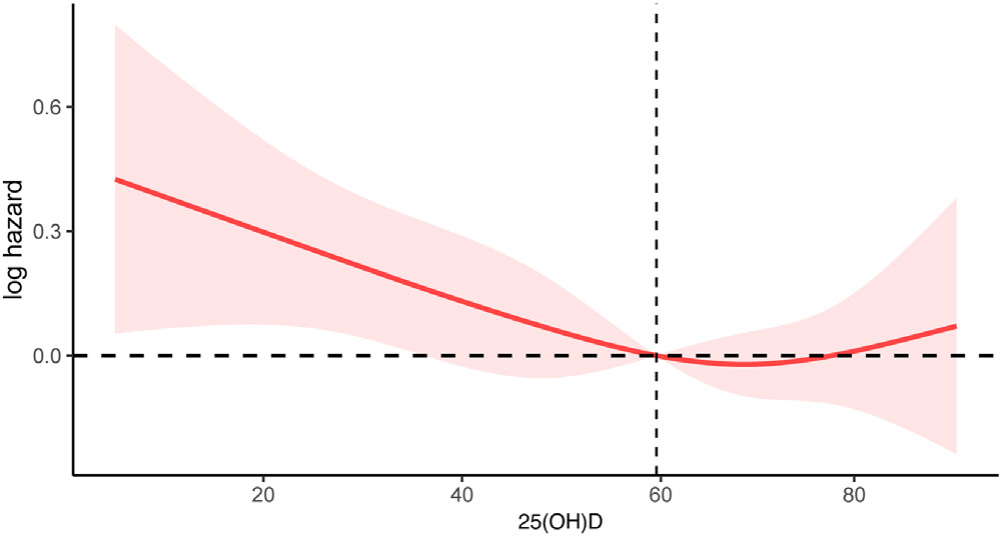
Weighted restricted cubic spline model for the associations between 25(OH)D and all-cause mortality. The weighted restricted cubic spline model was adjusted for sex, age, eth, BMI, smoke, history of cardiovascular disease, hypertension, diabetes, Hb, ALT, AST, Tb, ALP, Albumin, Creatinine, UA, BUN, Phosphorus, Calcium, HDL-c, LDL-c, eGFR, UACR, Dietary Vitamin D, CRP.

As shown in Fig. 4, the weighted restricted cubic splines revealed that the non-linear association between 25(OH)D and all-cause mortality was not statistically significant after adjustment for several potential confounders (p for nonlinearity > 0.05). Furthermore, the authors observed an inverse “J”-shaped linear relationship between 25(OH)D levels and the risk of all-cause mortality, whereby lower 25(OH)D levels were associated with an increased risk of all-cause mortality. A negative association was observed between 25(OH)D levels and all-cause mortality.

### Subgroup and sensitivity analysis

**Fig. 5:**
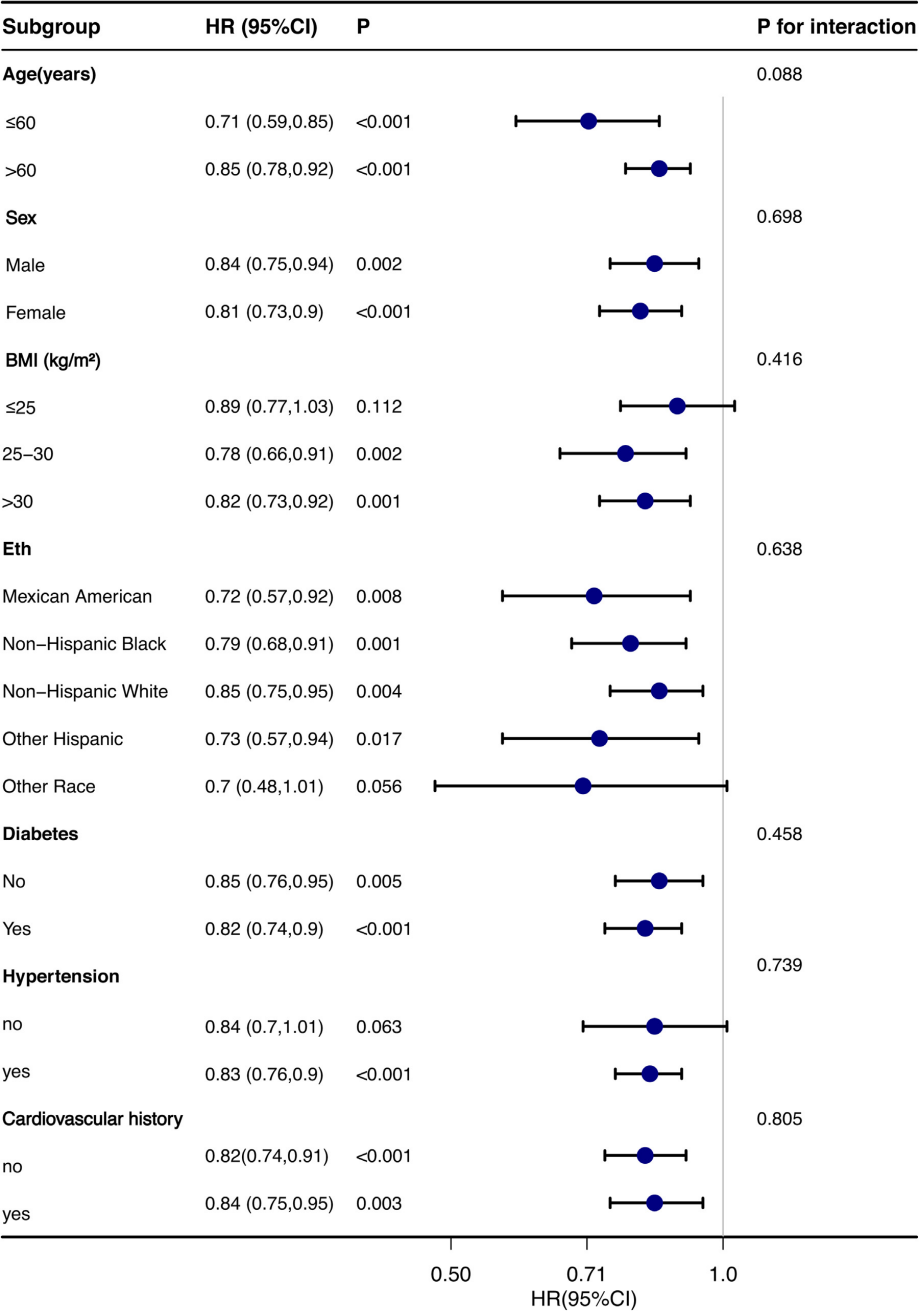
Forest plot for subgroup analysis of association between 25(OH)D and all-cause mortality.

Subgroup analyses were performed to investigate whether demographic characteristics and comorbidities accounted for the association between 25(OH)D and all-cause mortality, as shown in Fig. 5. The results remained consistent across the subgroups of age (< 60 years vs. ≥ 60 years), sex (male vs. female), BMI (< 25, 25‒30, ≥ 30), race, history of hypertension (yes or no), history of diabetes (yes or no), and history of cardiovascular disease, with no significant interaction with 25(OH)D (all p-values for interaction > 0.05). Notably, the association between 25(OH)D and all-cause mortality was not statistically significant in patients with BMI ≤ 25, other ethnic groups, or those without hypertension (p > 0.05). Sensitivity analyses excluding participants on dialysis yielded similar results to the primary analysis, increasing the robustness of the present findings (Table 4).

**Table 4:** Association between 25(OH)D and all-cause mortality among patients with CKD after excluding participants undergoing dialysis within the past 12 months (n= 2621).

25(OH)D	Crude Model		Model 1		Model 2	
	HR (95% CI)	p-value	HR (95% CI)	p-value	HR (95% CI)	p-value
Per SD increment	0.91 (0.84, 0.98)	0.012	0.80 (0.74∼0.87)	<0.001	0.85 (0.77∼0.93)	0.001
Categories						
Q1	Reference	Reference	Reference			
Q2	0.82 (0.66, 1.01)	0.066	0.79 (0.63∼0.98)	0.035	0.9 (0.7∼1.16)	0.414
Q3	0.81 (0.66, 1.01)	0.057	0.61 (0.48∼0.76)	<0.001	0.71 (0.54∼0.93)	0.014
Q4	0.71 (0.57, 0.89)	0.002	0.59 (0.47∼0.74)	<0.001	0.7 (0.54∼0.91)	0.008
p for trend	0.024	<0.001	0.003			

Crude Model: Unadjusted.

Model 1: Adjust for sex, age, eth, BMI, history of cardiovascular disease, hypertension and diabetes.

Model 2: Adjust for sex, age, eth, BMI, smoke, history of cardiovascular disease, hypertension, diabetes, Hb, ALT, AST, Tb, ALP, Albumin, Creatinine, UA, BUN, Phosphorus, Calcium, HDL-c, LDL-c, eGFR, UACR, Dietary Vitamin D, CRP.

## Discussion

In this nationally representative cross-sectional study, the authors observed a significant association between 25(OH)D levels and both all-cause and CVD mortality. Specifically, lower concentrations of 25(OH)D were associated with an increased risk of mortality. These findings remained consistent across various stratified and sensitivity analyses. Furthermore, the weighted restricted cubic splines analysis revealed that there was no statistically significant non-linear association between 25(OH)D and all-cause mortality after adjusting for multiple potential confounders (p for nonlinearity > 0.05). Additionally, the authors observed a linear relationship with an inverse “J”-shaped pattern between 25(OH)D levels and the risk of all-cause mortality. It should be noted that there are few longitudinal data on the association between 25(OH)D and mortality in patients with CKD. However, a small observational study involving 168 patients with CKD showed that 25(OH)D was predictive of death and progression of kidney disease.^14^ Observational studies have shown a significant association between lower levels of 25(OH)D and the progression of kidney disease.^15^^,^^16^ Vitamin D deficiency has been suggested as a prognostic factor for CKD progression and mortality. Robust cohort studies have consistently demonstrated that reduced circulating 25(OH)D levels are indicative of an increased risk of all-cause mortality in pre-dialysis CKD patients.^17^^,^^18^ These findings align closely with the outcomes observed in the present study.

Vitamin D synthesis is initiated in the skin by the conversion of 7-dehydrocholesterol to provitamin D3 by UV radiation, followed by its conversion to vitamin D3 by thermal processes. Vitamin D3 then enters the bloodstream, binds to vitamin D-binding protein, and reaches the liver for metabolism to 25-hydroxyvitamin D3. In the kidney, further metabolic pathways convert 25-hydroxyvitamin of D3 to 1.25-dihydroxyvitamin of D3. Importantly, vitamin D deficiency is common in patients with CKD, even in the early stages, and is more pronounced than in the general population. This may be due to several factors, including reduced 1α-hydroxylase activity as a result of reduced renal units and tubular dysfunction.^19^^,^^20^

Multiple mechanisms have been proposed to explain the association between 25(OH)D deficiency and the prognosis of patients with CKD, including regulation of cell proliferation,^20^ differentiation, immune modulation, bone metabolism, blood pressure control via renin regulation,^21^ and alterations in the inflammatory response associated with atherosclerosis.^22^ Within the renal sphere, there is a connection between vitamin D signaling and the morphology of podocytes, which results in safeguarding against disease progression. Additionally, vitamin D and its analogs have a positive effect on glomerular sclerosis and interstitial fibrosis by means of immunomodulatory and anti-inflammatory reactions, as well as inhibition of the renin-angiotensin system.^23^

In this study, the deficiency of 25(OH)D correspondingly increased mortality due to CVD. The potential mechanisms are as follows: Firstly, 25(OH)D may have an association with Fibroblast Growth Factor 23 (FGF23), where serum levels of FGF23 considerably increase in the presence of renal impairment, resulting in up-regulation of FGF23 and subsequent reduction in 1-α-hydroxylase activity. This reduction hinders the reabsorption of phosphorus in the renal tubes and restricts the production of 1.25-dihydroxyvitamin D. Increased levels of FGF23 have been associated with left ventricular hypertrophy, CVD-related death, and nephropathy progression in individuals suffering from chronic kidney disease.^24^

Furthermore, the present study showed an association between low 25(OH)D levels and proteinuria, which may also contribute to reduced 25(OH)D concentrations.^25^ Notably, proteinuria is an independent risk factor for cardiovascular disease and mortality in patients with CKD.^26^ Observational studies have consistently reported that vitamin D deficiency is associated with proteinuria, reduced glomerular filtration rate, and accelerated progression of CKD.^27^ In this cohort, the Q1 group had a significantly higher UACR compared to other groups, supporting the notion that more severe vitamin D deficiency is associated with a higher incidence of proteinuria. In addition, animal studies have suggested that vitamin D has beneficial effects on proteinuria, glomerular structure maintenance, and fluid regulation in various models of kidney disease.^28^ Vitamin D plays a crucial role in maintaining podocyte health by preventing epithelial-mesenchymal transition and inhibiting renin gene expression and inflammation.

Despite the results of this study indicating that low 25(OH)D concentration is a risk factor for mortality in patients with CKD, there is still a lack of consensus in the literature regarding the role of 25(OH)D in CKD and its optimal threshold. Maintaining adequate serum 25(OH)D levels is critical for the prevention of osteoporosis and cardiovascular disease and for preserving immune system function in CKD patients. Several studies have shown that vitamin D treatment can not only ameliorate but also reverse podocyte injury, particularly in 1.25-(OH)D3-deficient animal models.^29^ Impaired renal function can lead to impaired metabolism and activation of vitamin D, further reducing serum 25(OH)D levels. Therefore, maintaining adequate serum 25(OH)D levels is essential for the well-being of CKD patients.^30^

Interestingly, the present study also found that African Americans/Blacks had higher rates of vitamin D deficiency compared to other racial groups, which is consistent with previous research.^31^^,^^32^ This observation may potentially explain the disproportionately high prevalence of many cardiometabolic diseases associated with low vitamin D levels in African Americans.^31^ Notably, there are well-documented ethnic differences in vitamin D metabolites.^25^ The melanin-rich skin of darker-skinned individuals results in reduced absorption of UV-B necessary for vitamin D synthesis and consequently lower levels of 25(OH)D; however, the authors did not observe a significant interaction between ethnicity and study outcome for 25(OH)D. Furthermore, African Americans have an increased risk of all-cause mortality and cardiovascular disease associated with chronic kidney disease compared with European Americans.^33^ Whether this association is independent or influenced by the vitamin D-oxidative stress/inflammation interaction remains unknown.

The present study has several strengths and limitations. The authors conducted analyses using a large, nationally representative sample and adjusted for demographic, examination, laboratory covariates, and dietary vitamin D intake to ensure robustness and generalizability of associations. This approach allowed us to reliably assess the associations between 25(OH)D levels and study outcomes. Sensitivity analyses and subgroup analyses further confirmed the robustness of these findings, with consistent results. Some limitations should also be acknowledged. First, despite conducting a comprehensive sensitivity analysis, the utilization of an observational design in this study precludes us from establishing a definitive causal relationship between 25 (OH) D and outcome events. Second, this study only controlled dietary vitamin D intake; it did not adjust for information on the use of vitamin D supplements or active vitamin D analogs. In addition, because this information was self-reported, potential measurement errors cannot be completely ruled out. Furthermore, the authors were unable to assess the impact of changes in 25(OH)D during visits for all-cause and CVD deaths because measurements were taken only once at baseline in NHANES 2007‒2018; repeat measurements in future studies may allow a more accurate estimation of associations. Finally, despite adjustment for a wide range of confounders in this analysis process, there remains the possibility that residual or unmeasured confounders may still be present. In addition, future studies are expected to further explore optimal vitamin D status, while investigating effective treatment strategies to improve clinical outcomes.

## Conclusions

In conclusion, the present results show an inverse J-shaped association between 25(OH)D and all-cause mortality and a linear association between lower 25(OH)D concentrations and increased risk of all-cause and CVD mortality. These results support the independent prognostic value of 25(OH)D for mortality in patients with CKD. Therefore, the authors recommend routine 25(OH)D testing in all CKD patients, including those with ESRD, to identify individuals at high risk who may benefit from preventive treatment.

## Ethical approval

Reporting of the study conforms to the STROBE statement along with references to the STROBE statement (https://strobe-statement.org/). All procedures involving human participants were approved by the Research Ethics Review Board of the National Center for Health Statistics (protocol numbers: 2005-06, 2018-01, 2011-17).

## Informed consent statement

Informed consent was obtained from all subjects involved in the study.

## Data availability

The dataset generated and analyzed for this study is available in the National Health and Nutrition Examination Survey (NHANES), https://www.cdc.gov/nchs/nhanes/ (accessed August 11, 2023).

## Authors’ contributions

Luohua Li was actively involved in formulating the research questions, designing analyses, interpreting data, writing and reviewing the manuscript, and approving the version. Jinhan Zhao participated in the formulation of the research questions, analytical design, data analysis, data interpretation and approved the final version. All authors have read and approved the final version of the manuscript and are accountable for all aspects of the manuscript.

## Conflicts of interest

The authors declare that they have no known competing financial interests or personal relationships that could have appeared to influence the work reported in this paper.
